# Representation of anatomy in online atlases and databases: a survey and collection of patterns for interface design

**DOI:** 10.1186/s12861-016-0116-y

**Published:** 2016-05-21

**Authors:** Melissa D. Clarkson

**Affiliations:** Department of Biological Structure, School of Medicine, University of Washington, Seattle, WA USA

**Keywords:** Atlas, Database, Interface, Ontology, Anatomy

## Abstract

**Background:**

A large number of online atlases and databases have been developed to mange the rapidly growing amount of data describing embryogenesis. As these community resources continue to evolve, it is important to understand how representations of anatomy can facilitate the sharing and integration of data. In addition, attention to the design of the interfaces is critical to make online resources useful and usable.

**Results:**

I first present a survey of online atlases and gene expression resources for model organisms, with a focus on methods of semantic and spatial representation of anatomy. A total of 14 anatomical atlases and 21 gene expression resources are included. This survey demonstrates how choices in semantic representation, in the form of ontologies, can enhance interface search functions and provide links between relevant information. This survey also reviews methods for spatially representing anatomy in online resources. I then provide a collection of patterns for interface design based on the atlases and databases surveyed. These patterns include methods for displaying graphics, integrating semantic and spatial representations, organizing information, and querying databases to find genes expressed in anatomical structures.

**Conclusions:**

This collection of patterns for interface design will assist biologists and software developers in planning the interfaces of new atlases and databases or enhancing existing ones. They also show the benefits of standardizing semantic and spatial representations of anatomy by demonstrating how interfaces can use standardization to provide enhanced functionality.

## Background

Developmental biology is a data-intensive science. During the last two decades the primary means of archiving and accessing experimental results has shifted from traditional printed publications to digital repositories and web sites [1, 2]. This transition was driven by the need to mange the rapidly growing amount of data describing embryogenesis, integrate heterogeneous data, represent this data within the context of space and time, and enable cross-species comparisons.

Development of the first digital atlas took place in the early 1990s. These authors constructed a 9-day mouse embryo in 3D using serial sections at histological resolution, with plans to later incorporate spatially-based gene expression data [3]. Since that time, many anatomical atlases and gene expression databases have been developed for a number of species, by both individual laboratories and multi-institution teams.

Development of these resources has required partnering with computer science and informatics researchers. Areas of partnership include not only designing interactive web-based tools and improving methods of image capture and analysis, but also developing standards for data integration. Efforts to standardize elements of research (such as gene nomenclature, experimental protocols, descriptions of phenotypes, and the organisms themselves) began decades before the introduction of digital repositories [4, 5]. But standards are particularly important for digital repositories because the ability of users to find information relevant to their needs—and to make sense of what they find—is determined by the quality and consistency of the data and its annotations.

The Gene Ontology (GO) is a prominent example of an effort to develop semantic standards for molecular biology. GO allows biologists to describe the role of gene products shared across eukaryotic organisms [6]. It is widely used to cluster results of large-scale differential gene expression studies into functional categories, and has an important role in representing and aiding the discovery of gene regulatory networks [7, 8].

The representation of anatomy often receives less attention than the representation of genes, but is crucial because studies of gene expression must document not only the genes studied but also the anatomical location(s) of the expression. These annotations are most useful when they represent anatomy in ways that are explicit, standardized, and can be understood by researchers without expert knowledge of the species represented.

Methods for curating information about the anatomy of model organisms have evolved in parallel with methods for disseminating experimental data. Traditional print-based atlases have long been used to document anatomy and standardize terminology for structures and developmental stages. Online atlases provide additional benefits because they (a) allow data such as high-resolution two-dimensional (2D) images, three-dimensional (3D) reconstructions, and movies to be shared, (b) can be updated frequently, and (c) can link to external resources or incorporate content maintained by other sites.

**Fig. 1:**
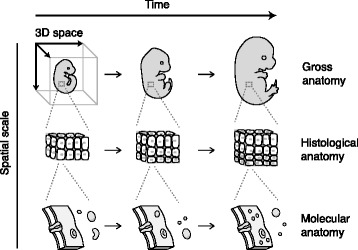
The components of a complete anatomical description. A complete description of an organism’s anatomy during development would account for three-dimensional structure at scales from gross to histological to molecular anatomy, and throughout the time of development

The task of representing an organism’s anatomy during development is inherently complex. As shown in Fig. 1, a complete description would account for three-dimensional structure at scales spanning gross, histological, and molecular anatomy, each throughout the time of development. Managing knowledge and data within this space-scale-time matrix presents a tremendous challenge. But it is also an opportunity to develop online atlases that not only provide anatomical descriptions, but also use anatomy as a framework for organizing and sharing data [9].

In order to develop atlases that will successfully serve as data portals for developmental biologists, research in a number of areas is crucial. These include development of web technologies for delivering volumetric image data over the web [10] and tools to support comparing data from disparate sources within a common spatial environment [11, 12]. Work related to gene expression data includes capture of quantitative expression data [13], mapping expression data to 3D graphics [14], visualization of data across time and space [15], and automating annotation of expression patterns with anatomical terms [16]. Atlases could also provide tools to aid researchers in analysis of their own data, such as feature for more precisely describing a specimen’s stage based on interpolating between reference stages [17, 18].

Atlases serving as data portals will require tightly integrated spatial and semantic representations as users alternate between image-based and term-based navigation and data retrieval. Therefore, in addition to research rooted in the fields of computer science and informatics, expertise is also needed from the fields of interaction design (to develop intuitive interfaces and effective visualizations) and knowledge representation (to provide semantic representations to enable data integration).

**Fig. 2:**
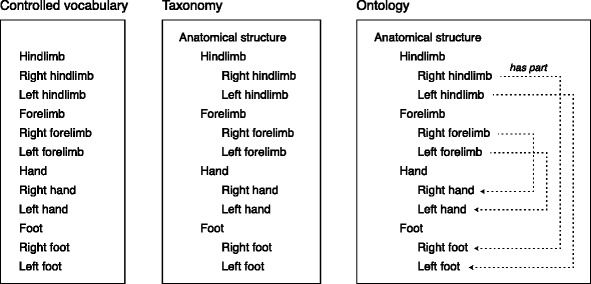
Levels of complexity in semantic representation. A controlled vocabulary is a list of defined terms. *A taxonomy* is a controlled vocabulary with a hierarchical structure formed by *is_a* relationships between pairs of terms. An *ontology* is a taxonomy with additional relationships, such as *part_of*

As shown in Fig. 2, the type of semantic representation determines the level of meaning captured within the representation. A controlled vocabulary is a list of terms within a specific domain. A taxonomy is a controlled vocabulary with hierarchical *is_a* relationships. An ontology is a taxonomy with additional relationships such as *has_part* and *develops_into*. The information provided by these relationships is necessary for developing atlases that link together data across space and time.

This article examines online resources for developmental biologists with an emphasis on semantic and spatial representation and interface design. I first survey 14 anatomical atlases and 21 gene expression resources for methods of anatomical representation. I then present a collection of patterns for interface design that demonstrate the variety of approaches used for anatomical representation, user interaction, and navigation with the atlases and databases. The purposes of this work are to (a) assist biologists and software developers in planning the interfaces of new atlases and databases or enhancing existing ones, and (b) demonstrate the benefits of standardizing semantic and spatial representations of anatomy.

## Results

### Anatomical atlases

For the purpose of this work, an anatomical atlas is defined as a resource that provides spatial representations of a body or region of a body, plus a set of anatomical terms which are associated with regions of the representations. I identified 14 online anatomical atlases describing organisms commonly used by developmental biologists.

Descriptions of each atlas are provided in Table 1. The six species represented (and number of atlases for each) are *Caenorhabditis elegans* (two), *Ciona intestinalis* (two), *Drosophila* (two), medaka (one), mouse (two), and zebrafish (four). Eight atlases have a spatial scope of the entire body, while others are limited to the brain (*Drosophila*, mouse), vasculature (medaka, zebrafish), midgut (*Drosophila*), or craniofacial skeleton (zebrafish). With the exception of one atlases for *C. elegans* and both *Drosophila* atlases, all atlases include a description of at least some developmental stages.

**Table 1:** Summary of anatomical atlases surveyed

Atlas	Description	Spatial scope	Developmental stages	URL, full project name, project leadership	Publication
*C. elegans*					
WormAtlas	A collection of resources including the SlidableWorm (for viewing annotated electron micrograph sections) and descriptions of individual neurons.	body	adult hermaphrodite, adult male, dauer larva	http://wormatlas.org From the laboratory of David Hall at Albert Einstein College of Medicine (Bronx, NY, USA).	[45]
OpenWorm Browser (Virtual Worm)	A 3D virtual reconstruction consisting of surface models of 680 cells.	body	adult only	http://browser.openworm.org A collaboration between WormBase and OpenWorm.	[46]
*Ciona intestinalis*					
ANISEED (“Anatomy” section)	Illustrations of ascidian embryos at selected stages, annotated with cell names.	body	egg through adult	http://www.aniseed.cnrs.fr Ascidian Network for In Situ and Embryological Data A collaboration among ascidian researchers led by Patrick Lemaire at the CRM (Montpellier, France).	[47] [48]
FABA	Confocal micrographs for standardizing developmental stages.	body	zygote through hatched larva	http://tunicate-portal.org/faba/1.4/top.html Four-dimensional Ascidian Body Atlas From the laboratory of Kohji Hotta at Keio University (Yokohama, Japan).	[49]
*Drosophila*					
Flygut	Description of the *Drosophila* midgut based on anatomy, histology, and expression patterns of reporter transgenes.	midgut	adult only	http://flygut.epfl.ch This atlas complements a publication from Bruno Lemaitre’s group at EPFL (Lausanne, Switzerland).	[50]
Virtual Fly Brain	Virtual sections from a reference brain, with anatomical regions delineated.	brain	adult only	http://www.virtualflybrain.org Members of the Virtual Fly Brain team are from the University of Edinburgh (Edinburgh, Scotland) and the University of Cambridge (Cambridge, England).	[51]
Medaka					
Medaka Blood Vessel Atlas	Annotated illustrations of the vasculature of embryos.	vasculature	mid-embryonic stages	http://www.shigen.nig.ac.jp/medaka/medaka_atlas Work by Misato Fujita and Sumio Isogai. Hosted online by the National BioResource Project Medaka (Japan).	[52] [53]
Mouse					
Allen Developing Mouse Brain Reference Atlas	Histological sections of brain with anatomical regions delineated.	brain	four embryonic stages, four post-natal stages	http://developingmouse.brain-map.org/static/atlas Produced by the Allen Institute for Brain Sciences (Seattle, WA, USA).	[54] [55]
e-Mouse Atlas	3D reconstructions of embryos (some with anatomical regions delineated), histological sections, and a guide to embryological stages.	body	all embryonic stages, three post-natal stages	http://www.emouseatlas.org/emap/ema/home.html also known as the Edinburgh Mouse Atlas Project led by Duncan Davidson and Richard Baldock within the Medical Research Council Human Genetics Unit and the University of Edinburgh (Edinburgh, Scotland).	[56] [57] [58] [59] [60] [61] [3]
Zebrafish					
FishFace	Graphics of fluorescently-labeled chondrocytes, osteoblasts, and bone matrix in the first two pharyngeal arches.	craniofacial skeleton	pharyngula through adult	https://www.facebase.org/fishface/home Created by the laboratory of Charles Kimmel at the University of Oregon (Eugene, OR, USA) as part of the FaceBase Consortium (USA).	[62]
FishNet	Virtual sections from optical projection tomography (OPT) scans (with selected sections annotated).	body	pharyngula through adult	http://www.fishnet.org.au Produced by Robert Bryson-Richardson and Peter Currie at Monash University (Clayton, VIC, Australia).	[63]
Interactive Atlas of Zebrafish Vascular Anatomy	Fluorescent angiograms as movies and annotated diagrams.	vasculature	pharyngula through larva	http://zfish.nichd.nih.gov/Intro%20Page/intro1.html Produced by Brant Weinstein’s group at the National Institutes of Health (Bethesda, MD, USA).	[64]
ZFAP	Graphics from the FishNet atlas within a viewer that shows three orthogonal planes.	body	pharyngula through adult	http://zebrafish.anatomyportal.org Zebrafish Anatomy Portal From Robert Bryson-Richardson’s group at Monash University (Clayton, VIC, Australia).	[65]
Zebrafish Atlas	Histological sections and a virtual slide viewer. Slides from two developmental stages are annotated.	body	larva through adult	http://bio-atlas.psu.edu/zf From the laboratory of Keith Cheng at Penn State College of Medicine (Hershey, PA, USA).	[66]

Table 2 lists the types of anatomical representations used within each atlas. Types of graphics included confocal micrographs, transmission electron micrographs (TEMs), histological sections, 3D reconstructions from histological sections, 3D surface models, illustrations, and brightfield movies. Six of the atlases stated that a controlled vocabulary or ontology was used as a source of terms for anatomical structures.

**Table 2:** Anatomical representations within the anatomical atlas

	Types of graphics^a^	Controlled vocabulary or ontology for anatomy
*C. elegans*		
WormAtlas	• TEMs • illustrations • DIC micrographs • fluorescence micrographs • confocal micrographs (as movies of volumes) • various movies, including 3D reconstructions from ssTEMs	not stated
OpenWorm Browser (Virtual Worm)	• 3D surface models	not stated
*Ciona intestinalis*		
ANISEED (“Anatomy” section)	• illustrations	*Ciona* Developmental Ontology
FABA	• confocal micrographs (as virtual sections and volumes) • brightfield movies	not stated^b^
*Drosophila*		
Flygut	• schematic illustrations • fluorescence micrographs • 3D surface models	not stated
Virtual Fly Brain	• confocal micrographs (as virtual sections) • 3D surface models of neurons	*Drosophila* Anatomy Ontology^c^
Medaka		
Medaka Blood Vessel Atlas	• illustrations • confocal microangiographs	not stated
Mouse		
Allen Developing Mouse Brain Reference Atlas	• histological sections with illustration overlays	Developing Mouse Brain Atlas ontology
e-Mouse Atlas	• 3D reconstructions of histological sections (as virtual sections and volumes) • histological sections • OPT scans (as volumes) • illustrations of stages	EMAP anatomy ontology
Zebrafish		
FishFace	• confocal micrographs (as projections) • OPT scans (as volumes)	Zebrafish Anatomy Ontology and [67]
FishNet	• OPT scans (as virtual sections and volumes)	not stated
Interactive Atlas of Zebrafish Vascular Anatomy	• illustrations • confocal microangiographs (as projections and volumes)	reference list on website
ZFAP	• OPT scans (as virtual sections and volumes)	Zebrafish Anatomy Ontology
Zebrafish Atlas	• histological sections	term list on website

^a^ Abbreviations: *DIC* differential inference contrast, *TEM* transmission electron micrograph, *ssTEM* serial section transmission electron micrograph, *OPT* optical projection tomography

^b^ The FABA established the developmental stages used in the *Ciona* Developmental Ontology

^c^ The vocabulary from the Insect Brain Name Working Group [68] was incorporated into the *Drosophila* Anatomy Ontology as part of the development of the Virtual Fly Brain atlas

### Gene expression atlases and databases

For this work, a gene expression atlas or database is defined as a resource that combines evidence of gene expression with a representation of the anatomical region of the expression. Because this work emphasizes spatial representations, resources consisting only of microarray data were excluded. I identified 21 resources for gene expression.

Descriptions of each atlas or database for gene expression are provided in Table 3. The eight species represented (and number of resources for each) are *Caenorhabditis elegans* (two), chicken (one), *Ciona intestinalis* (one), *Drosophila* (four), medaka (one), mouse (eight), *Xenopus* (two), and zebrafish (two). The spatial scope for 18 of the resources is the entire body. Others are limited to brain, nervous system, or urogenital system (each for the mouse). All resources include at least one developmental stage.

**Table 3:** Summary of gene expression atlases and databases surveyed

Atlas or database	Description	Spatial scope	Developmental stages	URL, full project name, project leadership	Publication
*C. elegans*					
Expression patterns for *C. elegans* promoter::GFP fusions	Database of expression patterns of transgenic animals with promoter::GFP fusions	body	embryo through adult	http://gfpweb.aecom.yu.edu/index A project of the British Columbia *C. elegans* Gene Expression Consortium	[69]
WormBase (“WormMine” tool)	Community repository of molecular and genetic data from the literature, submissions, and collaborating projects	body	embryo through adult	http://www.wormbase.org An international consortium of researchers, based at Caltech (Pasadena, CA, USA)	[70] [46]
Chicken					
GEISHA	Community repository of in situ hybridization data acquired from high-throughput screens and the literature	body	egg through first six days of development	http://geisha.arizona.edu/geisha *Gallus* Expression in Situ Hybridization Analysis Hosted by Parker Antin’s group at the University of Arizona (Tuscaon, AZ, USA)	[71] [72] [73]
*Ciona intestinalis*					
ANISEED (“Gene Expression & Function” section)	Community repository of expression data from the literature, submissions, and collaborating projects	body	egg through adult	http://www.aniseed.cnrs.fr Ascidian Network for In Situ and Embryological Data A collaboration among ascidian researchers led by Patrick Lemaire at the CRM (Montpellier, France)	[47] [48]
*Drosophila*					
BDGP expression patterns	Database of in situ hybridization patterns	body	all embryonic stages	http://insitu.fruitfly.org/cgi-bin/ex/insitu.pl Berkeley Drosophila Genome Project From the laboratory of Susan Celniker at the Lawrence Berkeley Laboratory (Berkeley, CA, USA)	[74] [75] [76]
FlyBase (“QuickSearch” tool)	Community repository of molecular and genetic data from the literature and direct submissions	body	egg through adult	http://flybase.org From an international consortium of *Drosophila* researchers	[77] [78] [79]
FlyExpress	A tool for searching expression patterns using images data from BDGP and Fly-FISH	body	all embryonic stages	http://www.flyexpress.net From the laboratory of Sudhir Kumar at Arizona State University (Tempe, AZ, USA)	[40] [80]
Fly-FISH	Database of in situ hybridization patterns	body	early embryonic stages and third instar larva	http://fly-fish.ccbr.utoronto.ca From the laboratory of Henry Krause at the University of Toronto (Toronto, Ontario, Canada)	[81]
Medaka					
MEPD	Database of expression patterns for genes (in situ hybridization) and regulatory sequences (fluorescent reporters)	body	egg through adult	http://mepd.cos.uni-heidelberg.de/mepd Medaka Expression Pattern Database A project within the Medaka Genome Initiative, from the laboratory of Joachin Wittbrody at University of Heidelberg Heidelberg, Germany)	[82] [83] [84]
Mouse					
Allen Developing Mouse Brain Atlas (“AGEA” section)	Atlas of spatially correlated expression patterns derived from in situ hybridizations on histological sections	brain	four embryonic stages (E11.5, 13.5, 15.5, 18.5), four post-natal stages (P4, 14, 28)	http://developingmouse.brain-map.org/agea/show Anatomic Gene Expression Atlas of the Allen Developing Mouse Brain Atlas Produced by the Allen Institute for Brain Sciences (Seattle, WA, USA)	[85] [86] [87] [36]
EMBRYS	Database of in situ hybridization patterns, using whole mounts	body	three embryonic stages (E9.5, 10.5, 11.5)	http://embrys.jp/embrys/html/MainMenu.html *Note: no longer available online* From the laboratory of Hiroshi Asahara at the Systems BioMedicine Laboratory of the National Research Institute for Child Health and Development (Tokyo, Japan)	[88] [89]
EMAGE	Community repository of molecular and genetic data from the literature and direct submissions	body	all post-implantation stages	http://www.emouseatlas.org/emage/home.php e-Mouse Atlas of Gene Expression Led by Duncan Davidson and Richard Baldock within the Medical Research Council Human Genetics Unit at the University of Edinburgh (Edinburgh, Scotland)	[56] [90] [91] [58] [92] [93]
Eurexpress	Database of in situ hybridization patterns, using histoloogical sections	body	one embryonic stage (E14.5)	http://www.eurexpress.org/ee From a consortium of European researchers	[94]
GXD	Community repository of expression data from the literature, direct submissions, and collaborating projects	body	all embryonic stages, postnatal	http://www.informatics.jax.org/gxd Mouse Gene Expression Database A Mouse Genome Informatics resource from Jackson Laboratory (Bar Harbor, ME, USA)	[95] [96] [97] [98]
GENSAT	Database of in situ hybridization patterns and data from transgenic mice with EGFP reporter genes	nervous system	one embryonic stage (E15.5), postnatal (P7), adult	http://www.gensat.org/index.html Gene Expression Nervous System Atlas From the laboratory of Nathaniel Heinz at The Rockefeller University (New York, NY, USA)	[99] [100]
GenePaint	Database of in situ hybridization patterns, using histological sections	body	three embryonic stages (E10.5, 14.5, 15.5), postnatal (P7), adult	http://genepaint.org Led by Gregor Eichele at the Max Planck Institute of Biophysical Chemistry (Göttingen, Germany)	[101]
GUDMAP	Community repository of expression data from the GUDMAP consortium	urogenital system	mid-embryonic development through adult	http://www.gudmap.org GenitoUrinary Molecular Anatomy Project From an international consortium of researchers	[102] [103] [104]
*Xenopus*					
Xenbase	Community repository of expression data from the literature, submissions, and collaborating projects	body	all stages through adult	http://www.xenbase.org Led by Peter Vize at the University of Calgary (Calgary, Canada)	[105] [106]
XenMARK expression patterns	Database of in situ hybridization patterns, using whole mounts	body	32-cell stage through tadpole	http://genomics.crick.ac.uk/apps/XenMARK From the laboratory of Michael Gilchrist at the MRC National Institute for Medical Research (London, UK)	[38]
Zebrafish					
GEMS	Database of in situ hybridization patterns	body	gastrula through hatching	http://bio-imaging.liacs.nl/liacsgems.html *Note: no longer available online* Gene Expression Management System Produced by the Imagery & Media Group at Leiden University (Leiden, Netherlands)	[107]
ZFIN	Community repository of molecular and genetic data from literature and direct submissions	body	zygote through adult	http://zfin.org Zebrafish Model Organism Database (also known as the Zebrafish Information Network) Based at the University of Oregon (Eugene, OR, USA)	[108] [109] [110]

Table 4 lists the anatomical representations used within each gene expression atlas or database. Types of graphics documenting expression patterns include brightfield micrographs showing a colorimetric assay for expression in both histological sections and whole mounts, confocal micrographs, and 3D models with the region of expression highlighted. At least eight of the resources rely primarily on submissions of data from the community, and therefore various types of graphics are presented across different database entries. All resources use anatomical terms to describe regions of expression. However, only 14 use terms from controlled vocabularies or ontologies. The other resources appear to use project-specific vocabularies.

**Table 4:** Anatomical representations within the gene expression atlases and databases

Atlas or database	Types of graphics^a^	Controlled vocabulary or ontology for anatomy^b^
*C. elegans*		
Expression patterns for *C. elegans* promoter::GFP fusions	• fluorescence micrographs • confocal micrographs (as projections and volumes) • DIC micrographs with fluorescence overlays	project-specific vocabulary
WormBase (“WormMine” tool)	• various types submitted • Virtual Worm model with regions of gene expression highlighted	*C. elegans* Gross Anatomy Vocabulary *C. elegans* Development Vocabulary
Chicken		
GEISHA	• various types submitted	project-specific vocabulary
*Ciona intestinalis*		
ANISEED (Expression Data section)	• various types submitted	*Ciona* Developmental Ontology
*Drosophila*		
BDGP expression patterns	• brightfield micrographs (whole mounts, colorimetric assay)	*Drosophila* Anatomy Ontology
FlyBase (“QuickSearch” tool)	–	*Drosophila* Anatomy Ontology
FlyExpress	• graphics from BDGP and Fly-FISH	vocabularies from BDGP and Fly-FISH
Fly-FISH	• confocal micrographs	project-specific vocabulary
Medaka		
MEPD	• brightfield micrographs (whole mounts, colorimetric assay for gene expression) • fluorescence micrographs (for regulatory element expression)	MFO
Mouse		
Allen Developing Mouse Brain Atlas (“AGEA” section)	• brightfield micrographs (histological sections, colorimetric assay) • standardized 3D brain models with regions of expression highlighted	Allen Developing Mouse Brain Atlas ontology
EMBRYS	• brightfield micrographs (whole mounts, colorimetric assay) • AERO images (whole mounts, colorimetric assay)	project-specific vocabulary
EMAGE	• various types submitted	EMAP anatomy ontology
Eurexpress	• brightfield micrographs (histological sections, colorimetric assay)	EMAP anatomy ontology
GXD	• various types submitted	EMAP anatomy ontology; MA
GENSAT	• brightfield micrographs (histological sections, colorimetric assay) • confocal micrographs	project-specific vocabulary
GenePaint	• brightfield micrographs (histological sections, colorimetric assay)	project-specific vocabulary
GUDMAP	• various types submitted	EMAP anatomy ontology
*Xenopus*		
Xenbase	• various types submitted	XAO
XenMARK expression patterns	• brightfield micrographs (whole mounts, colorimetric assay)	project-specific vocabulary
Zebrafish		
GEMS	• confocal micrographs	DAOZ
ZFIN	• various types submitted	ZAO

^a^ Abbreviations: *DIC* differential inference contrast, AERO images are a series of 2D images captured at 2-degree intervals [88]

^b^ Abbreviations: *DAOZ* Developmental Anatomy Ontology of Zebrafish, *MFO* Medaka Fish Anatomy and Development Ontology, *XAO Xenopus* Anatomy Ontology, *ZAO* Zebrafish Anatomy Ontology

### Semantic representation: Controlled vocabularies and ontologies

Table 5 lists the controlled vocabularies and ontologies used in the resources surveyed. This survey revealed that a total of 12 vocabularies or ontologies are used. Each includes terms relevant to adult anatomy, developmental anatomy and stages, or both. Seven organisms are represented (*C. elegans, C. intestinalis, Drosophila,* medaka, mouse, *Xenopus,* and zebrafish). As shown in Table 5 (third column, indicated with superscript), each vocabulary and ontology was developed by one of the groups creating an atlas or database included in Tables 1 and 3.

**Table 5:** Controlled vocabularies and ontologies relevant to this survey

Vocabulary or ontology	Domain	Used by this atlas or database	URL, full name	Publication
*C. elegans*				
*C. elegans* Gross Anatomy Vocabulary	• developmental and adult anatomy, including individual cells	WormBase^a^	http://bioportal.bioontology.org/ontologies/WB-BT also known as the *C. elegans* Cell and Anatomy Ontology	[111]
*C. elegans* Development Vocabulary	• developmental stages • time points	WormBase^a^	http://bioportal.bioontology.org/ontologies/WB-LS	–
*Ciona intestinalis*				
*Ciona intestinalis* Anatomy and Development Ontology	• developmental and adult anatomy, including individual cells • developmental stages	ANISEED^a^	http://bioportal.bioontology.org/ontologies/CIINTEADO	[47] [48]
*Drosophila*				
*Drosophila* Anatomy Ontology	• developmental and adult anatomy • includes vocabulary from the Insect Brain Name Working Group	VFB FlyBase^a^	http://bioportal.bioontology.org/ontologies/FB-BT also known as the *Drosophila* Gross Anatomy Ontology	[68] [27] [112]
*Drosophila* Development Ontology	• developmental stages • cycles of nuclear division	FlyBase^a^	http://bioportal.bioontology.org/ontologies/FB-DV	_
Medaka				
MFO	• developmental and adult anatomy • developmental stages	MEPD^a^	http://bioportal.bioontology.org/ontologies/MFO Medaka Fish Anatomy and Development Ontology	[83]
Mouse				
MA	• adult anatomy	GXD^a^	http://bioportal.bioontology.org/ontologies/MA Adult Mouse Anatomy, also known as the Mouse Adult Gross Anatomy Ontology	[22]
Allen Developing Mouse Brain Atlas ontology	• developmental anatomy	Allen Developing Mouse Brain Atlas^a^	http://help.brain-map.org/display/api/Atlas+Drawings+and+Ontologies	[55]
EMAP	• developmental anatomy • includes vocabulary from the GUDMAP consortium	e-Mouse Atlas^a^ GXD GUDMAP	http://bioportal.bioontology.org/ontologies/EMAP e-Mouse Atlas Project anatomy ontology	[21] [113] [114]
*Xenopus*				
XAO	• developmental and adult anatomy • developmental stages	Xenbase^a^	http://bioportal.bioontology.org/ontologies/XAO *Xenopus* Anatomy Ontology, also known as the *Xenopus* Anatomy and Development Ontology	[24] [115]
Zebrafish				
DAOZ	• developmental anatomy • developmental stages	GEMS^a^	http://bio-imaging.liacs.nl/liacsontology.html Developmental Anatomy Ontology of Zebrafish	[116]
ZFA	• developmental and adult anatomy • developmental stages	ZFIN^a^	http://bioportal.bioontology.org/ontologies/ZFA Zebrafish Anatomy Ontology, also known as the Zebrafish Anatomy and Development Ontology	[117]

^a^ The vocabulary or ontology was developed by the group constructing this atlas or database

Several atlases and gene expression resources demonstrate how the relationships within ontologies can contribute to the usefulness of interfaces by enhancing search functions or providing links between relevant information:

- *Part relationships:* EMAGE [19] and GXD [20] are databases for gene expression in the mouse, and both have a search function that accepts a term for an anatomical structure and returns genes expressed in that structure. These databases make use of the part hierarchies in the EMAP anatomy ontology [21] and Adult Mouse Anatomy (MA) [22] to return results annotated with either the term entered by the user or the parts of that structure. For example, a search for genes expressed in “eye” will return genes annotated with “eye”, “retina”, and “lens vesicle”.
- *Developmental relationships:* Searches for gene expression in an anatomical structure can be expanded by including structures linked by developmental relationships. The Xenbase gene expression database [23] provides an option to include successor and predecessor structures in search results. These relationships are provided by the *Xenopus* Anatomy Ontology (XAO) [24]. Developmental relationships also provide a way for users to navigate an atlas or database along developmental pathways. GUDMAP [25] is a database of gene expression in the mouse urogenital system. It employs the *derives_from* and *differentiates_into* relationships as links between the tissue summary pages with include gene expression data. This enables a user examining data annotated with “early distal tubule” to follow the *differentiates_into* relationship to data annotated with “renal distal tubule”.
- *Structural relationships:* Structural relationships allow a resource to present knowledge specific to an anatomical context. For example, the Virtual Fly Brain [26] provides an interface that uses the *has_presynaptic_terminal_in* and *has_postsynaptic_terminal_in* relationships for nerves in the *Drosophila* Anatomy Ontology [27]. For example, when viewing the medulla in the atlas, lists are generated for neurons with presynaptic and postsynaptic terminals the medulla. Producing the list of neurons requires two types of knowledge from the ontology: (a) which anatomical structures are *part_of* the medulla, (b) each neuron that *has_presynaptic_terminal_in* or *has_postsynaptic_terminal_in* those parts.

Ontologies also provide an opportunity to link resources to each other. For example, each anatomical term in the Zebrafish Anatomy Portal (ZFAP) [28] is linked to a page in ZFIN [29] that defines the term and provides ontological relationships.

### Spatial representation: 2D and 3D graphics

The atlases and databases in this survey demonstrate several ways that spatial representation of anatomy can be enhanced in a web-based resource:

- *Graphics of developmental stages:* The process of development can be studied only if it is represented in ways that are sufficiently rich in detail and reasonably standardized — a challenge as old as embryology itself [30]. Web-based atlases are able to represent spatial structure and time-based processes in way that traditional print-based resources cannot. For example, the Four-dimensional Ascidian Body Atlas (FABA) [31] defines stages for *C. intestinalis* with confocal image stacks and time-lapse movies, and the e-Mouse Atlas provides 3D reconstructions of embryos for many Theiler stages. Standardization of annotation for developmental stages is necessary for sharing data among laboratories, and atlases provide an easy way to access stage descriptions.
- *High-resolution histological sections:* Glass histology slides have long been used for studying histology. Virtual slides are created by scanning and digitizing glass slides, and the experience of using a microscope is simulated through web applications that allow zooming and panning of the image. Virtual slides are provided by the Zebrafish Atlas [32] and e-Mouse Atlas [33].
- *Visual representation of ontological terms:* Web-based resources provide an environment in which to link semantic and spatial representations of anatomical structures. For example, the Allen Developing Mouse Brain Atlas [34] and the Virtual Fly Brain [26] provide linked term-and-graphic windows that provide a view of both the ontology used and structures annotated with those terms. The Zebrafish Anatomy Portal (ZFAP) [28] provides a search function that takes a term from the Zebrafish Anatomy Ontology and returns planes of reconstructions from optical projection tomography (OPT) scans labeled with the term.
- *Correlating spatial data among specimens and experiments:* For gene expression patterns to be useful, they must be annotated in a way that allows users to find genes that are expressed in regions of interest and to study co-expression patterns. Four methods are used to annotate expression patterns within the resources surveyed: (a) The first method is manual annotation of each specimen using terms from a controlled vocabulary or ontology. This method enables only text-based queries, and will be inaccurate where expression patterns do not correspond to the borders of defined anatomical regions. (b) The second approach relies on computational annotation and preserves the spatial nature of the data. In this approach, the expression pattern of each specimen is registered to a stage-matched standard volume through spatial warping enabling spatial queries to be performed across the dataset. This is used by the e-Mouse Atlas of Gene Expression (EMAGE) [19] (as described in [35]) and Allen Developing Mouse Brain Atlas [34] (as described in [36]). (c) A third approach, used by XenMARK [37], relies on a manual annotation process in which specimen expression patterns are drawn onto stage-specific schematic diagrams [38]. This method avoids the computational complexity of spatial warping, but allows spatial searches from the schematics. (d) A fourth approach is to compute similarity scores between pairs of images, instead of mapping to a stage-specific standard. This method is used by FlyExpress [39] on sets of 2D images of *Drosophila* embryos that are uniformly oriented and assayed under the same conditions [40].

### Patterns for interface design

In order to document and generalize the approaches for conveying information about anatomy that are used in these atlas and databases, I compiled a set of patterns. Patterns are reusable solutions to design problems, and are of great interest in interface design [41]. The patterns I have identified focus on graphic representation, user interaction, and navigation.

**Fig. 3:**
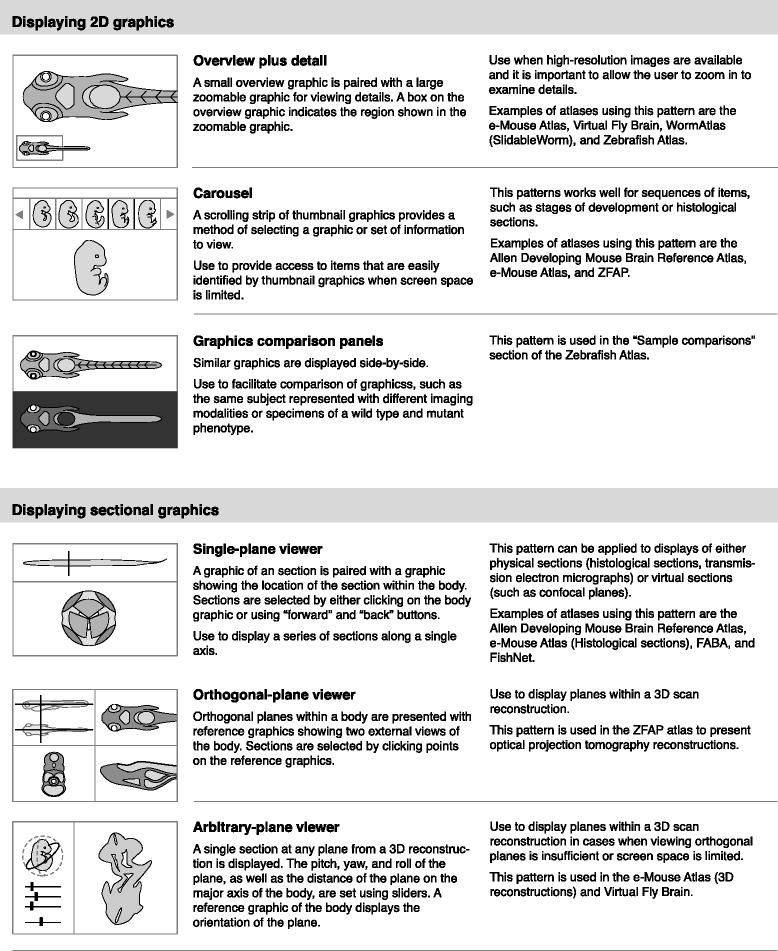
Interface design patterns for displaying 2D graphics and sectional graphics

**Fig. 4:**
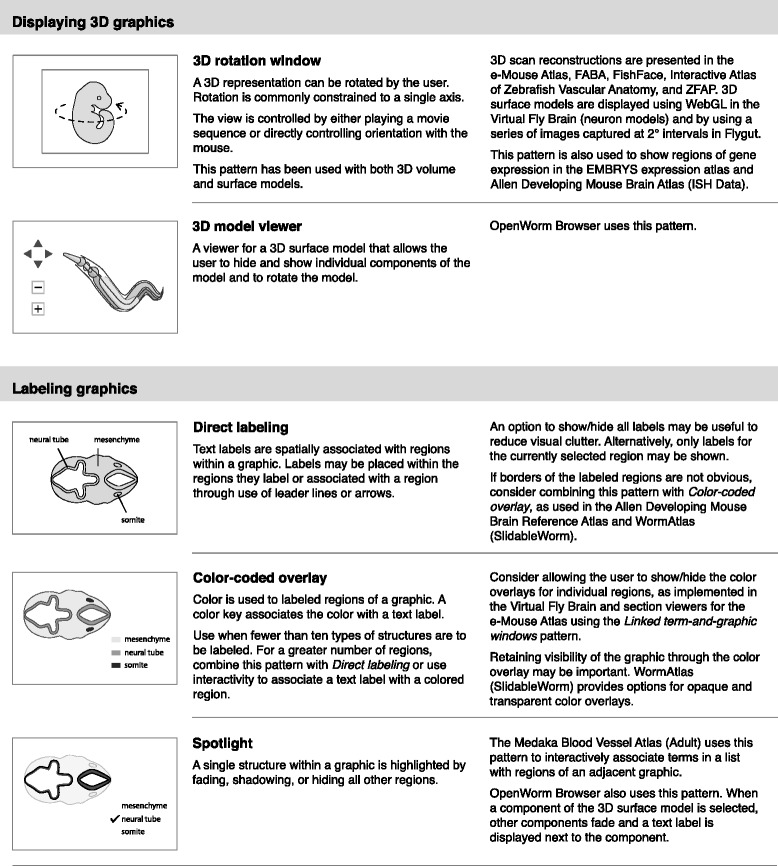
Interface design patterns for displaying 3D graphics and labeling graphics

**Fig. 5:**
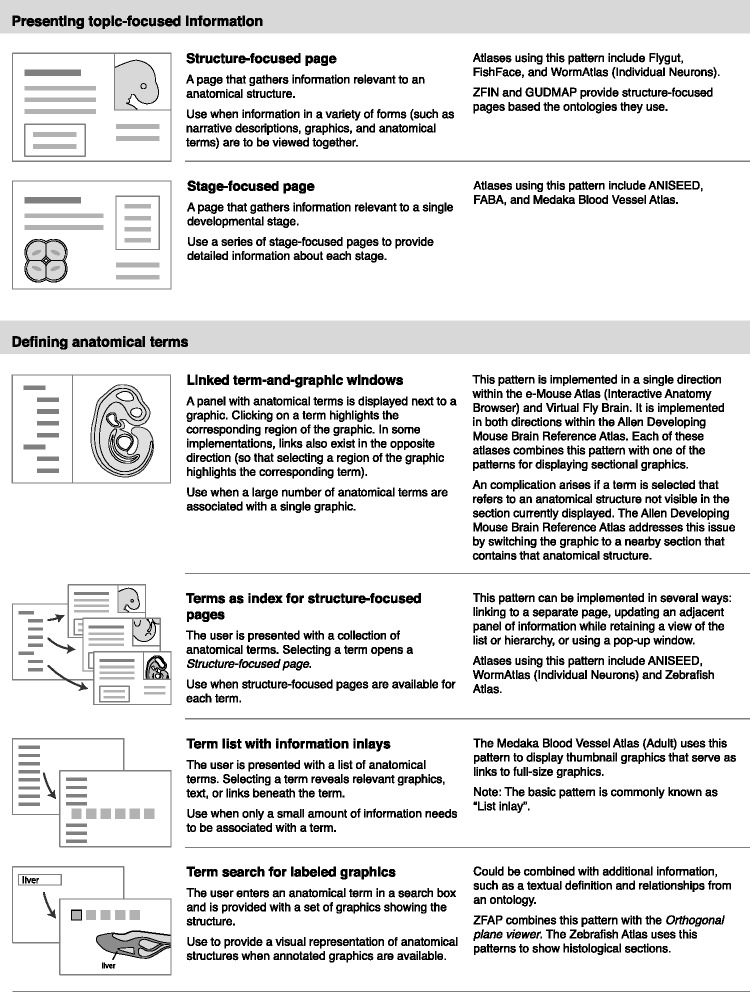
Interface design patterns for presenting topic-focused information and defining anatomical terms

**Fig. 6:**
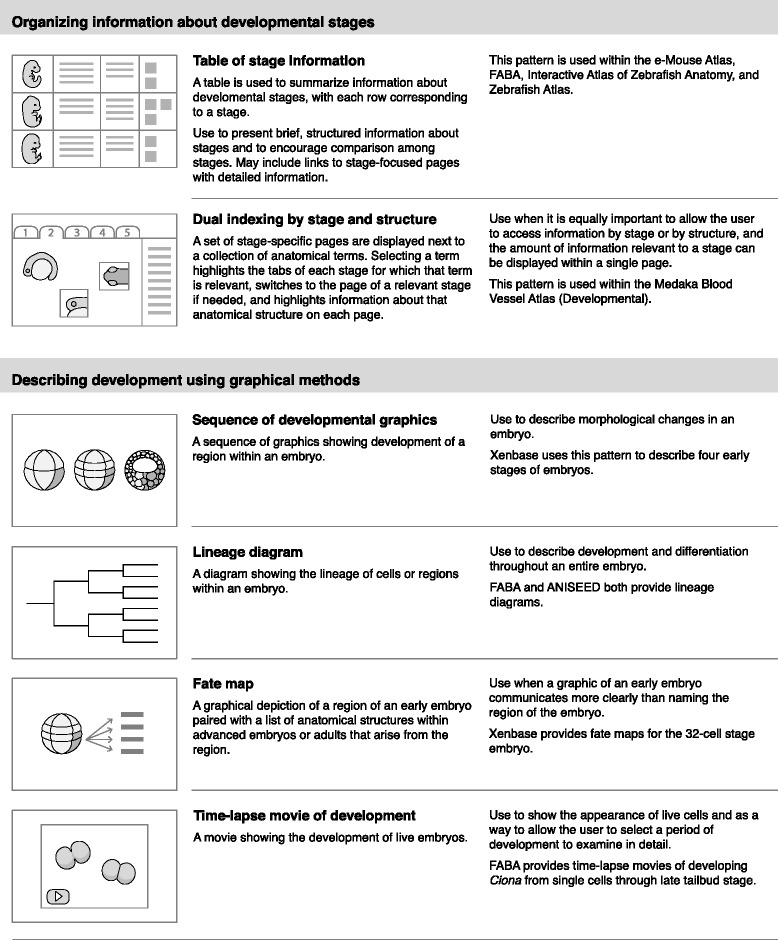
Interface design patterns for organizing information about developmental stages and describing development using graphical methods

From the anatomical atlases I identified a total of 23 design patterns, shown in Figs. 3, 4, 5 and 6. These patterns provide methods for displaying graphics, integrating semantic and spatial representations, and organizing atlas information. They are grouped into eight categories:

- Displaying 2D graphics
- Displaying sectional graphics
- Displaying 3D graphics
- Labeling graphics
- Presenting topic-focused information
- Defining anatomical terms
- Organizing information about developmental stages
- Describing development using graphical methods

Each pattern has a title, description, examples of use, and simple pictorial representation. For example, one pattern in the category of “Displaying 2D graphics” is “Overview plus detail.” This pattern pairs a small overview graphic with a large zoomable graphic. A box on the small graphic indicates the region shown in the zoomable graphic. This pattern can be used when high-resolutions graphics are available and it is important to allow the user to zoom into details. This pattern is used within the e-Mouse Atlas [33], Virtual Fly Brain [26], WormAtlas (SlidableWorm) [42], and Zebrafish Atlas [32].

**Fig. 7:**
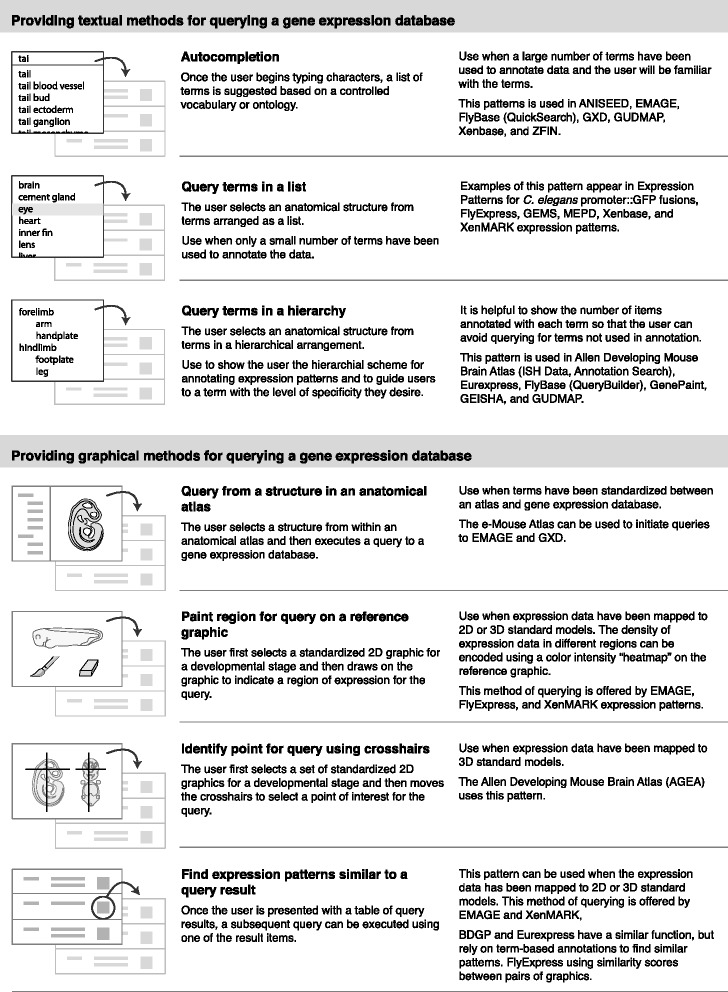
Interface design patterns for textual and graphical methods of querying a gene expression database

**Fig. 8:**
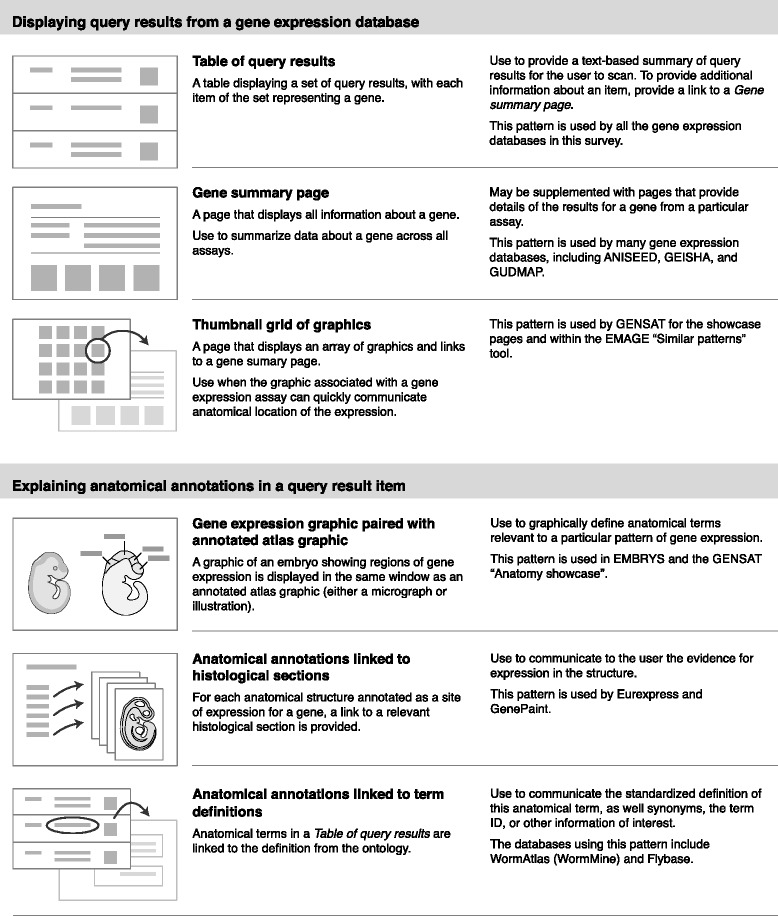
Interface design patterns for displaying query results from a gene expression database and explaining anatomical annotations

From the gene expression atlases and databases I identified 13 patterns that provide methods for querying databases to find genes expressed in anatomical structures and display the results. These patterns are shown in Figs. 7 and 8, and are grouped into four categories:

- Providing textual methods for querying a gene expression database
- Providing graphical methods for querying a gene expression database
- Displaying query results from a gene expression database
- Explaining anatomical annotations in a query result item

A few of the patterns that I present here have been previously identified in other pattern collections. In these cases I have retained the names given by previous authors (for example, “Overview plus detail”, “Autocompletion”, and “Thumbnail grid“) [41].

## Discussion

This collection of design patterns can be used as a catalyst for conversations between biologists and software developers. Because they provide a user-focused perspective, they can support discussions of methods for exploring and retrieving anatomically-based information and can serve as building blocks for interface specifications.

The patterns also help to clarify how interface functionality is constrained by the investment in semantic and spatial standardization. For example, one of the most intuitive ways to query a gene expression pattern database is by specifying a point or region on a standardized image, but this requires that the expression data have been mapped to a spatial standard.

This work documents the variety of ways anatomical information has been communicated in online atlases and databases. Part of this variability is due to differences in the types of data available, purposes of the resources, and the characteristics of the organism. But it also reflects the large number of design solutions that are possible. Because this survey did not include resources presented as downloadable software, there are likely to be additional patterns for representing anatomy.

### Using the patterns to support user needs

**Fig. 9:**
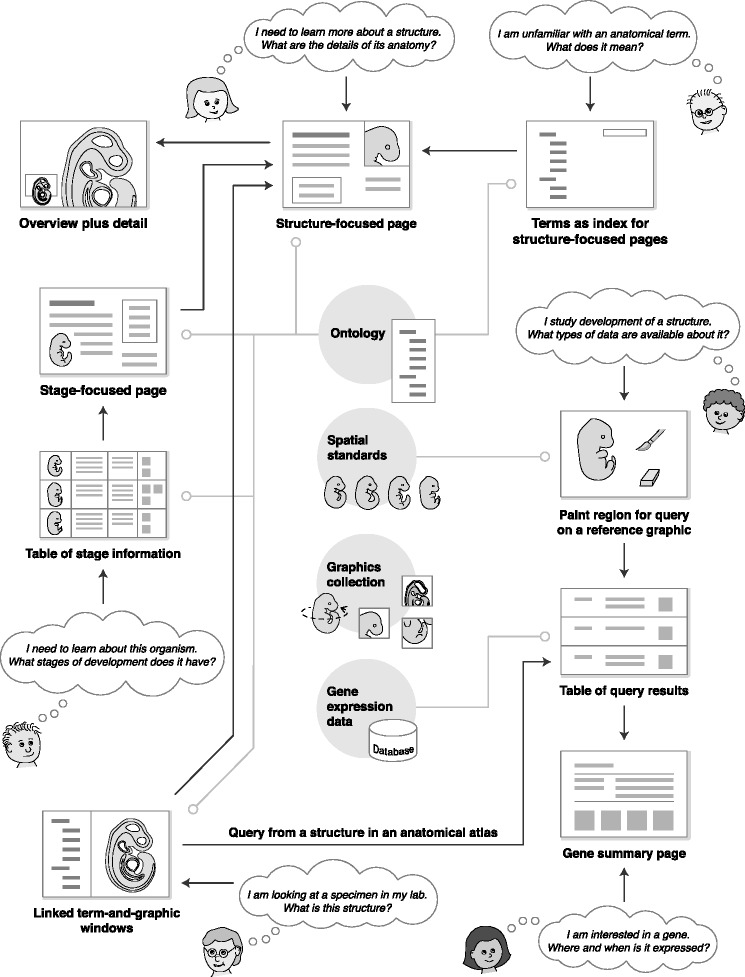
Scenario for an online resource combining an anatomical atlas with gene expression data. Questions by users (in thought clouds) serve as entry points to pages using the design patterns. Arrows represent links between pages using different patterns. Four types of information assets (an ontology, spatial standards, graphics collection, and gene expression data) are shown in the center. Lines extending from the information assets indicate some of the patterns that incorporate those assets

When applied to developing or expanding a particular online resource, this work should be considered in the context of two important considerations: “What are the information needs of the users?” and “What information assets are available to use in building the resource?” The patterns act as intermediaries between the users’ questions and the information assets. Figure 9 provides a scenario that uses ten of the patterns in an imagined resource. This resource provides an anatomical atlas and gene expression database, and uses information assets including an ontology (serving as the semantic standard), spatial standards, a graphics collection, and gene expression data. In this scenario, user needs include obtaining information about the meaning of anatomical terms and finding data related to development of a particular anatomical structure.

### Designing for new types of data

Looking to the future, standardized spatial representations will be key to communicating and integrating new types of data. Emerging techniques for “spatially resolved omics” [43] provide high-throughput measurements of gene expressions while preserving detailed spatial information. For example, microtomy sequencing provides gene expression data from individual cryosections of a specimen. Recent work with zebrafish embryos extends this approach by analyzing data from embryos sectioned along orthogonal body axes using image reconstruction algorithms to produce 3D expression patterns—a technique called RNA tomography (or tomo-seq) [44].

The detailed spatial data produced by large-scale gene expression studies are unlikely to correspond to traditionally defined regions of anatomy. Therefore, standardized spatial representations of model organisms will need to serve as a mediator between the data collected by laboratories and the users of community databases. In this scenario, investigators will not annotate their data with semantic annotations. Rather, they will map their data to standard spatial representations. The role of semantic representations will be to serve as references to regions of the models and logical links between models at different stages or at different spatial scales. This will integrate disparate data at the level of spatial representation, while preserving the usefulness of semantic representations for filtering, searching, and browsing data.

## Conclusions

This work highlights issues important for the continued evolution of online resources for developmental biology. If resources are to be effective in helping researchers to locate data relevant to their questions and to generate hypotheses, they must provide a structure that allows researchers to navigate within the space-scale-time matrix depicted in Fig. 1, as well as to explore homologous structures across different species. This next generation of resources—perhaps better described as web portals—will provide access to not only information from different laboratories stored within a single repository, but to information distributed across different repositories. These web portals (and perhaps networks of interlinked web portals) will rely on tightly integrated semantic and spatial representation, using anatomy as a framework for data integration, organization and navigation.

As model organism communities move toward the goal of building a comprehensive understanding of development, the role of these web portals is crucial. They will serve both to document collective knowledge from previous work and to provide the infrastructure that enables future work. Achieving this vision will require not only advances in web and imaging technology, but careful consideration of semantic and spatial representation and research to design usable and intuitive interfaces. In order for these tools to meet the needs of biologists, biologists must partner with computer science, informatics, and design researchers.

## Methods

The atlases and databases surveyed in this work were identified through keyword searches of the Science Direct, Scopus, and PubMed databases. The keywords used were *atlas* or *database* in combination with *Arbacia, Caenorhabditis elegans, chick, chicken, Ciona, Danio rerio, Drosophila, fly, frog, Gallus gallus, medaka, mouse, Mus musculus, nematode, Oryzias latipes, rat, Rattus norvegicus, sea squirt, sea urchin, Strongylocentrotus, Xenopus,* or *zebrafish*. Several additional atlases and databases were identified based on URL links within these resources. Keyword searches were performed on 26 July 2014 and 9 Oct 2015.

Resources included in this survey are (a) described in a peer-reviewed journal article, (b) publicly available, (c) delivered on the web without requiring download, and (d) in English. In addition, a resource must have been available on at least one of the dates of testing (26 July 2014, 4 Aug 2014, 6 Sept 2014, and 9 Oct 2015). If a resources consists of both online material and downloadable material, only the online material was included in this survey. Two resources became unavailable during the course of this project (EMBRYS and GEMS). These are included in the survey, but their unavailability is noted with their URLs in Table 3.

Resources were excluded if they are primarily (a) databases of microarray data, (b) collections of figures from journal articles, (c) collections of graphics or movies with little or no annotation of anatomical structures, or (d) textbook-like resources with limited navigation structure. This work is limited to model organisms, and therefore resources for human anatomy and development are excluded.

All resources were viewed using operating system Mac OSX 10.9.5 with Java 1.8.0 and the Firefox 41.0 browser. Components provided as self-signed Java applications were excluded from this survey due to security risks.

## Ethics approval and consent to participate

Not applicable.

## Consent for publication

Not applicable.

## Availability of data and material

This study did not generate datasets.

## Abbreviations

2D: two dimensional
3D: three dimensional
DIC: differential inference contrast
OPT: optical projection tomography
ssTEM: serial section transmission electron micrograph
TEM: transmission electron micrograph
